# iSupport, an online training and support program for caregivers of people with dementia: study protocol for a randomized controlled trial in India

**DOI:** 10.1186/s13063-018-2604-9

**Published:** 2018-05-08

**Authors:** Kala M. Mehta, Dolores Gallagher-Thompson, Mathew Varghese, Santosh Loganathan, Upasana Baruah, Katrin Seeher, Diana Zandi, Tarun Dua, Anne Margriet Pot

**Affiliations:** 1 Department of Epidemiology and Biostatistics, University of California, San Francisco, CA USA; 2 Department of Psychiatry and Behavioral Sciences, Stanford University School of Medicine, Stanford, CA USA; 3 Geriatric Clinic and Services, Department of Psychiatry, National Institute of Mental Health and Neurosciences, Bengaluru, India; 4 World Health Organization (WHO), Geneva, Switzerland

## Abstract

**Background:**

Dementia has a huge physical, psychological, social and economic impact upon caregivers, families and societies at large. There has been a growing impetus to utilize Internet interventions given the potential scalability, and presumed cost-effectiveness and accessibility. In this paper, we describe the design of a randomized controlled trial (RCT) aiming to study the impact of online self-help programs on caregivers of people with dementia in India. The experimental group will receive an interactive training and support program and the comparison group will receive an education-only e-book. It will be among the first online support intervention RCTs for a mental health condition in a lower-middle income country.

**Methods and design:**

Two hundred and eight participants are expected to be recruited via several strategies (email, Internet and social media, telephone and face-to-face) starting in the Bangalore region of India. The inclusion criteria for participation in the trial are: (1) being 18 years or older, (2) being a self-reported caregiver of a person with dementia, (3) self-report that a family member has a diagnosis of dementia (AD8 ≥ 2), and experience caregiver distress (≥ 4 on a 1-item burden scale ranging from 1 to 10 *or* ≥ 4 or < 20 on the Center for Epidemiologic Study-Depression (CES-D) scale (10-item) or ≥ 4 or < 15 on the Generalized Anxiety Disorder Scale (7-item).

The intervention group will be offered iSupport, an online self-help training and support program, enabling a personalized education plan with a maximum of 23 lessons. These modules present a range of topics from “what is dementia?” to “dealing with challenging behaviors like aggression.” The comparison group will receive an education-only e-book containing similar content. The outcomes of this trial are: caregiver burden as measured by the 22-item Zarit Burden Scale, depressive symptoms, anxiety symptoms (primary outcomes), quality of life, person-centered attitude, self-efficacy and mastery (secondary outcomes).

**Discussion:**

Based on the findings of this trial, we will examine the potential use and scale up of iSupport for caregiver distress in India. This style of online self-help programs could be expanded to other regions or countries or to other suitable caregiver groups.

**Trial registration:**

Clinical Trials Registry—India (CTRI), ID: CTRI/2017/02/007876.

**Electronic supplementary material:**

The online version of this article (10.1186/s13063-018-2604-9) contains supplementary material, which is available to authorized users.

## Background

Dementia is a global public health priority since the disease has a huge impact on caregivers, family and society in physical, psychological, social and economic terms [1, 2]. Currently, there are 47.5 million people with dementia worldwide, a number that is due to triple by 2050 [2, 3]. Most people with dementia are cared for by family or other unpaid and untrained caregivers without any additional support, especially in lower-middle income countries [2, 4]. These caregivers often face numerous stressors, such as time pressures, changed behaviors and financial stress which can be challenging for them to cope with and balance with demands from work and family [2]. These stressors often lead to significant health problems such as depression, anxiety and physical problems for caregivers themselves [5, 6].

Research, mostly carried out in high-income countries, shows that training and support might help reduce psychological distress in caregivers of people with dementia. For example, support groups for caregivers of people with dementia have a beneficial impact on diverse caregiver outcomes, including feelings of burden, mental health, depressive symptoms, and, for example, the quality of the relationship with the person with dementia, for women in particular [7]. In these support groups, caregivers share their experiences of caring; learning about dementia and its impact on daily life. They learn about available resources and are trained to better cope with the person with dementia and the care situation.

A large body of evidence, also mostly carried out in high-income countries, shows that multicomponent interventions have a beneficial impact on caregivers of people with dementia. Some multicomponent interventions are customized and tailored to the specific situation of the caregivers and their needs [8–10]. They include, for example, skills training for caregivers, psycho-education, planning pleasant activities, changes in the environment, increasing social support, teaching techniques to better care for themselves, physical training, home care and case management [5, 10–13].

In the last decade, there has been a growing interest in the use of information and communications technologies and the Internet for people with dementia and their caregivers, including online training and support programs [14]. One line of research has been to create caregiver interventions specifically for Internet delivery. A potential reason is scalability, which is important and urgent because of the demographic changes. Internet-based approaches are also attractive because caregivers can access the program at a convenient time in the privacy of their own home. They do not have to travel to a clinic or physician appointment which may induce stigma, because they often do not consider themselves as the ones who need care, and it does not require arranging alternate care for their family member, or transportation [15].

A few trials in high-income countries showed promising results of Internet-based training and support programs for caregivers of people with dementia, with a reduction of caregiver depressive symptoms, anxiety symptoms and perceived stress, although the full potential of these programs has not been reached yet [16–19].

Though one often thinks of caregiver distress in high-income country contexts, but it is, in fact, an even larger issue for lower-middle income countries, like India [20, 21]. There are only a few formal skilled nursing facilities, retirement homes and other residential care options for older people in India [22, 23]. Most people with dementia (at least 95%) are cared for at home by their family only [4, 24, 25]. The need for training and support services for people with dementia and their caregivers far outpace the health and social services and personnel that exist. As the population of India (over 1.3 billion) ages, this will be a growing problem.

Using Internet-based methods for caregiver training and support may also be promising in lower-middle income countries, in countries with a high number of Internet users like India. The number of Internet users in India is estimated at more than 460 million and Internet penetration is rapidly increasing [26].

The development of training and support for caregivers is emphasized as a strategic priority in the World Health Organization (WHO) action plan on dementia and on aging and health, both approved by the WHO’s 194 member states. This is especially the case for lower-middle income countries such as India [21, 27]. Therefore, the WHO has brought together a panel of international experts to develop “iSupport,” a self-help online training and support program aimed at relieving caregivers’ psychological distress.

In this article, we describe the design of an RCT in India to examine the effectiveness of iSupport.

## Methods and analyses

**Fig. 1:**
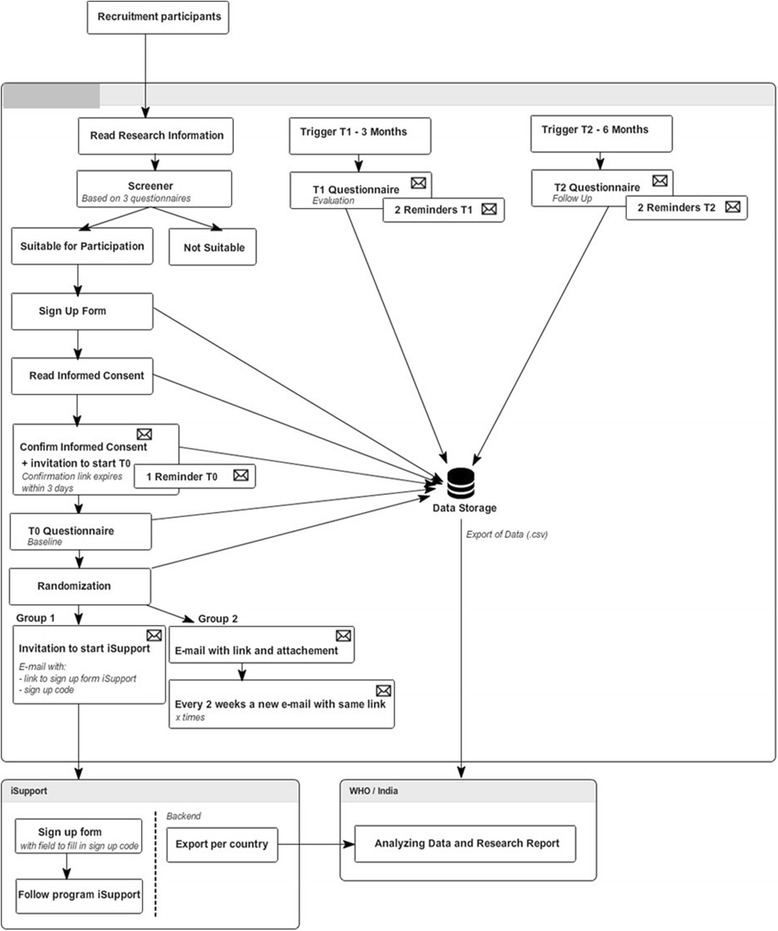
Flowchart of the iSupport Online Self-Help Program. T0 pre-measurement, T1 = first follow up 1–3 months post baseline, T2 second follow up: 2–6 months post T0

**Fig. 2:**
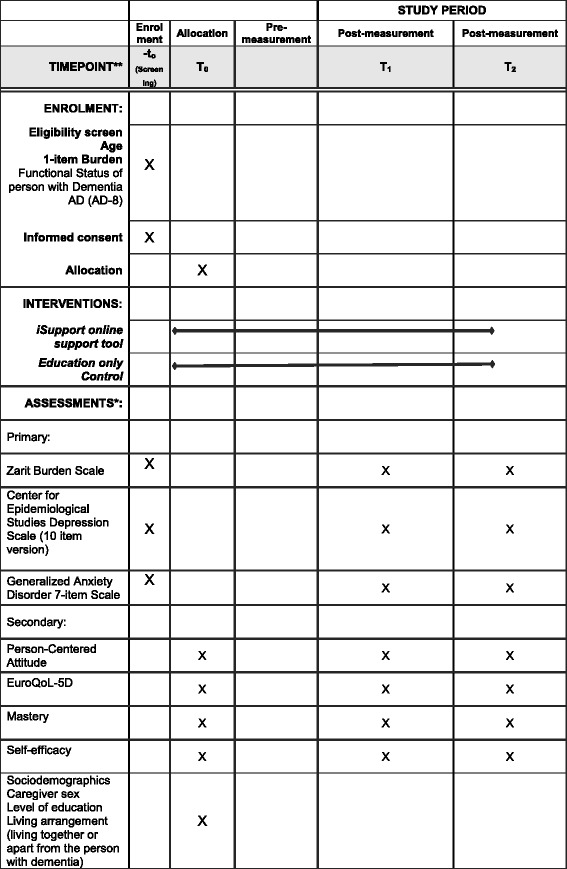
SPIRIT Flowchart of the iSupport online self-help program

The trial design is a two-group RCT to establish the effectiveness of an online self-help training and support program (iSupport) compared to an education-only comparison condition (EOC) which provides education about dementia and caregiving through an on-line e-book (Fig. 1). The objective is to examine the effect of this online self-help training and support program on depression and anxiety outcomes. The RCT will last 6 months in total, including pre measurement (T0), first follow up (T1, 3 months after T0) and second follow-up (T2, 3 months after T1). The timeline of assessments and measures are described in Fig. 2.

This trial is set in India, a lower-middle income country with a rapidly aging population and a high number of Internet users. National Institute for Mental Health and Neuro Sciences (NIMHANS), one of the premier institutes that provide neuropsychiatric services to older people in India, is executing the trial.

### Ethical approval

The study was approved by the Medical Ethics Committees of the World Health Organization (WHO) and the National Institute of Mental Health and Neurosciences (NIMHANS). The trial was registered at the Clinical Trials Registry—India (CTRI), protocol # CTRI/2017/02/007876.

### Recruitment

Participants are recruited using several strategies: social media outreach, telephone calls and face-to-face contact. On social media (Facebook, WhatsApp and Twitter), regular posts including a short video, electronic flyers and messages are posted to targeted groups that may have an interest in online training and support. In addition, email flyers are sent to NIMHANS staff, community-based organizations serving older people, and employees at information technology companies in the local Bangalore area (with permission). On-going publicity appears in Bangalore print media. Person-to-person recruitment strategies include the posting of flyers at NIMHANS; for example, in the waiting areas of specific clinics for people with dementia. In addition, family caregivers of people with dementia who are already in the NIMHANS or some other care systems involved in the recruitment for this study are personally invited to participate in the study. Beyond Bangalore, recruitment efforts are primarily through Alzheimer’s and Related Disorders Society of India (ARDSI) with chapters in 20 large cities throughout India and some specialists who are involved in the health care for people with dementia.

### Inclusion criteria

Participants are screened online to assess whether they are (1) aged 18 years and older, (2) are a self-reported caregiver of a person with dementia, (3) self-report that their family member has a diagnosis of Alzheimer’s disease or dementia (score ≥ 2 on the AD8) [28]. In addition, the caregiver should reside in India and have access to the Internet. For inclusion, the caregiver should experience some psychological distress, as indicated by some subjective burden (defined by a score of ≥ 4 on a 1-item burden scale ranging from 1 to 10) [29] or some symptoms of depression or anxiety (score ≥ 4 and < 15 on the Generalized Anxiety Disorder (GAD) Scale (7 items) [30] or ≥ 4 and < 20 on the Center for Epidemiologic Study-Depression (CES-D) scale (10 item) [31, 32]. If potential participants have a score of 20 or higher on the 10-item CES-D, and ≥ 15 on the GAD, caregivers are referred to the mental health services at NIMHANS or providers in their region. As this is an online intervention, and participants may be from all over India, no further referral or follow-up are given.

### Randomization

After screening, participants who fulfil the inclusion criteria are requested to provide informed consent to enrol in the study. Next, they are asked to fill out the questionnaires at baseline, after which randomization is carried out using an automated computer-generated random number allocation, stratified by sex and relationship to the care recipient (spouse/other) with a block size of 2. Participants are masked to their intervention status.

To randomize the participants, a table is filled with “real random” 0 or 1 codes preceding the start of the project, drawn with equal probability (50%) from the Random.org site [33]. This table is subsequently linked to the table with the accumulation of participant data. Immediately before randomization, the questionnaire script determines what block-condition (male/female; spouse/no spouse) applies to the participant. Next, with a block size of 2, there are two options:

- If a new block must be opened for the new participant: the participant’s condition will be determined by the “real random” value in the linked table. Thus, if the random value is 0, the participant’s condition will be 0 and if the random value is 1, the condition will be 1
- If a block is already open and partially filled (thus, with a block size of 2, with one other respondent), the new participant’s condition will be the other condition of the first participant. So, if the first participant has condition 0, the new participant’s condition will be 1, etc.

Baseline characteristics of individuals randomized to either of the two conditions will be compared to ensure that randomization was successful. If an individual that is enrolled in the trial drops out after enrollment, is lost to follow-up or discontinued from the study, their data will be analyzed using the intention-to-treat principle.

### iSupport: online self-help training and support program

**Fig. 3:**
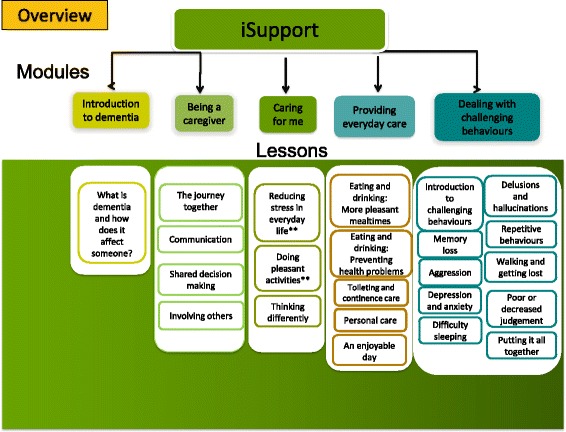
iSupport: online self-help training and support program

iSupport consists of information, skills training, and support for caregivers, using problem-solving and cognitive-behavioral therapy techniques, addressing the needs of caregivers (Fig. 3). The online program contains the following five themes: (1) what is dementia? (one lesson); (2) being a caregiver (four lessons); (3) caring for me (three lessons); (4) providing everyday care (five lessons); and (5) dealing with challenging behavior (ten lessons).

Each lesson presents a topic and is comprised of several interactive exercises rather than long pages with theoretical information only. Caregivers are given instant feedback when they provide their answers to the questions in the exercises. Caregivers can compose their own personalized education plan for which they can select any lesson they decide would be particularly pertinent. Figure 3 provides an overview of the lessons, summarizing the themes. An adapted version to the Indian context of the generic WHO field-testing version of Support was pilot tested for usability and acceptability by potential caregivers in India (*N* = 10). We will monitor adherence using the online support tool including time spent on the tool, per week. Internal to the support system are positive messages at the completion of lessons included to increase adherence.

### Comparison condition: education-only control

The comparison condition – education-only – is an e-book based on a brochure created for caregivers of people with dementia by Alzheimer’s Disease International in collaboration with the World Health Organization [34]. It contains information on the following topics: basic information about dementia; living with and caring for a person with dementia; practical tips on managing dementia; personal and emotional stress of caring; caring for yourself; help for you, the carer, and; what support tools are available through Alzheimer’s Disease International. It does not have an interactive component like iSupport to teach caregivers skills for managing their mood or techniques for coping with challenging behaviors in people living with dementia.

### Sample size calculation

Sample size calculation is guided by the primary outcome change in burden (ZBS) at 6 months after baseline. Alpha (two-tailed) was set at 0.05 power at 80%. A standardized effect size of .33 – a relevant effect for the Zarit Burden Scale (ZBS) [35] – was calculated using earlier findings from a recent trial which evaluates power/sample size for these studies [36]. Using Stata 12, minimum sample size for each group was identified as 80. To take account of study dropout we increase this number by 30% (*N* = 208; 104 per group).

### Outcomes

The primary outcomes are burden and symptoms of depression and anxiety (Table 1). Burden is measured by the 22-item Zarit Burden Scale [35]. Responses range from 0 (never) to 4 (nearly always). Depressive symptoms are measured using the modified version of the Center for Epidemiological Studies Depression scale (CES-D10): ten items measuring frequency of common depressive symptoms (e.g., sadness, hopelessness, sleep and appetite disturbances) in the past week. Response categories are: 0 (rarely or none of the time present) to 3 (most or all of the time present) [32]. Anxiety symptoms are measured using the General Anxiety Disorders scale (GAD-7), a 7-item measure rating the frequency of common anxiety symptoms (nervousness, worry, not being able to relax, being restless, annoyed or irritable, and feeling afraid that something awful will happen) in the past 2 weeks. Response categories are: 0 (not at all), 1 (several days), 2 (more than half the days) and 3 (nearly every day) [30].

**Table 1:** Primary and secondary outcome measures and study variables in the iSupport online support trial

Outcome measures in the randomized controlled trial (RCT) (number of items, response scale, total scale score range)						
Primary	Target of scale	Reliability and validity of scales	Cronbach’s alpha	Pre measurement (T0)	First Follow up (T1)	Second Follow-up (T2)
Zarit Burden Scale (22, 0–4, 0–88)^a^	Perceived stress of caregiving	Intra-class correlation coefficient (ICC): 0.88 to 0.89	Cronbach’s α = 0.82 to 0.93	X		X
CES-D10 Center for Epidemiological Studies Depression Scale (10 items, 0–3, 0–30)^b^	Depression	Test-retest reliability (r values = 0.41 to 0.70)	Cronbach’s α = 0.80 to 0.85	X	X	X
GAD Generalized Anxiety Disorder 7-item scale (7 items,0–3, 0–21)^c^	Anxiety	ICC = 0.83	Cronbach’s α = 0.92	X		X
Secondary						
EuroQoL-5D Descriptive system of health-related quality of life states consisting of 5 dimensions (mobility, self-care, usual activities, pain/discomfort, anxiety/depression).^d^	Quality of Life	ICC = 0.70	No overall Cronbach's α reported		X	X
Mastery (7 items, 1–4, 7–28)^e^	Perlin 7-item Mastery Scale	Principal component factor loadings ranging from − 0.47 to 0.76			X	X
Self-efficacy (1, 0–5, 0–40)^f^	RIS Self-efficacy Scale	*r* = 0.48 to 0.69	Cronbach’s α = 0.72 to 0.79			X
Confounders/modifying Variables						
Behavioral Problems RMBPC (6, 0–4, 0–24)^g^	Revised Memory and Behavioral Problems Checklist	Rs = 0.29, .31, .26; ps < .01 correlations between subscales and caregiver depression (CES-D) and burden (Caregiver Stress Scale)	Cronbach’s α values are: .75 for frequency and .76 for reaction for memory problems .82 and .77 for depression and .62 and .70 for disruptive behavior	X		X
Functional status of person with dementia AD^h^	Informant Questionnaire on Cognitive Decline in older people (AD8)	*r* = 0.75	Cronbach’s α = 0.84	X		X
Demographic variables caregiver’s age, caregiver sex, relationship to the person with dementia (spouse, adult child, or other family member), level of education, living arrangement (living together or apart from the person with dementia)				X		

^a^Zarit S, Reever KE, Back Peterson. Relatives of the impaired elderly: correlates of feelings of burden. The Gerontologist. 1980;20:649–655

^b^Björgvinsson T, Kertz SJ, Bigda-Peyton JS, McCoy KL, Aderka IM. Psychometric properties of the CES-D 10 in a psychiatric sample. Assessment*.* 2013;20: 429–436

Radloff LS. CES-D scale: a self report depression scale for research in the general populations. Applied Psychological Measurement*.* 1977;1:385–401

^c^Spitzer R, Kroenke K, Williams JBW, Lowe B. A brief measure for assessing generalized anxiety disorder. Arch Inern Med*.* 2006;166:1092–1097

^d^Brooks R, Rabin R, de Charro F (Ed.). The measurement and valuation of health status using EQ-5D: a European perspective: evidence from the EuroQol BIO MED Research Programme. Rotterdam: Kluwer Academic Publishers; 2003

^e^Pearlin LI, Schooler C. The structure of coping. Journal of Health and Social Behavior*.* 1978;19:2–21

^f^Steffen A, McKibbin C, Zeiss A, Gallagher-Thompson D, Bandura A. Revised Scale for Caregiving Self-Efficacy: reliability and validity studies. J Gerontol B Psychol Sci Soc Sci*.* 2002;57: P74–P86

^g^Teri L, Truax P, Logsdon R, Uomoto J, Zarit S, Vitaliano PP. (1992). Assessment of behavioral problems in dementia: The Revised Memory and Behavior Problems Checklist (RMBPC*).* Psychology and Aging*.* 7(4):622–31.

^h^Galvin JE, Roe CM, Powlishta KK, Coats MA, Muich SJ, Grant E, Miller JP, Storandt M, Morris JC. The AD8: a brief informant interview to detect dementia. Neurology. 2005; 65:559–564

^h^Galvian JE, Roe CM, Xiong C, Morris JC. Validity and reliability of the AD8 informant interview in dementia. Neurology. 2006;67:1942–1948

The secondary outcomes are caregiver quality of life, person-centered attitude, self-efficacy and mastery. Quality of life is measured by the EuroQol-5 Dimension (EQ-5D) [37]. Person-centered attitude is measured by a subscale within the Approaches to Dementia Questionnaire (ADQ) [38]. For measuring self-efficacy we use the RIS Eldercare Self-efficacy Scale [39]. Mastery is measured using the 7-item Pearlin Mastery Scale [40].

### Covariates

Several other variables are measured, such as the age of the caregiver, their sex, their relationship to the person with dementia (spouse, adult child, or other family member), level of education of the caregiver, living arrangement (living together or apart from the person with dementia).

All study data will be collected within the iSupport online tool using encryption for participant protection. Data are securely stored using a cloud-based encrypted platform. Data will only be accessed by project team members involved in data analyses. Confidentiality will be maintained before, during and after the trial. Study participants who discontinue use of the tool will be sent automatic weekly reminders to promote adherence. There is no external data monitoring board. The study team will take appropriate action in case any adverse effects are reported.

### Statistical analyses

Analyses will be conducted according to the intention-to-treat principle. Randomization efficacy will be assessed by examining characteristics in the iSupport condition compared to EOC and missing data and unbalance will be evaluated. Missing data on follow-up measurements will be imputed using multiple imputations. To examine the association between the experimental and comparison group on the primary and secondary outcomes, we will first calculate paired *t* tests and estimates of effect sizes will be calculated. By using multiple regression analyses, we can correct for possible confounders. With a “generalized estimating equations” (GEE) analysis the research question regarding the maintenance of the effectiveness of the intervention will be answered for primary and secondary outcomes in separate models [41]. Thus, all primary and secondary outcome measures will be analyzed using separate GEE models using each separate outcome measure as the dependent variable and an indicator for the intervention (the iSupport intervention compared to the education-only control) as the primary independent variable. Potential confounders will be added to these models. All analyses will be conducted using SPSS for Windows.

## Discussion

Dementia is a global public health priority. Especially in lower-resourced settings, a huge gap exists whereby there are not enough care providers and yet, there is a rapidly growing rate of dementia. Given this, risk reduction of dementia is important, as is the training and support for family and other caregivers to provide care to people who already have dementia or will have dementia. Family caregivers face significant challenges when providing care to people with dementia, putting them at greater risk for severe health problems, such as depression, anxiety and functional decline [27, 34, 42, 43].

In this RCT, we study the effectiveness of iSupport, an online training and support program. It is among the first of its kind adapted for a lower-resourced setting, delivered without the intervention of a health care professional, easily accessible via the Internet. In this trial, caregivers who use iSupport are compared to those who receive education only using the following outcomes: burden, and symptoms of depression and anxiety (primary outcomes); quality of life, person-centeredness, self-efficacy and mastery (secondary outcomes). The outcomes chosen for this trial have been used in several other trials related to Internet support and caregiver distress, internationally [14, 16–18].

There are some factors that may hinder participant inclusion for the trial. Firstly, online self-help training and the support program for caregivers are not commonly used and may not yet be readily accepted, particularly not by older caregivers in India. In general, receiving health information via the Internet in India is still relatively new. This may hamper the recruitment because caregivers tend not to automatically search for help on the Internet in general, which might also be exacerbated by the stigma related to dementia and to help-seeking [44]. This may also result in a relatively high dropout rate during the intervention period. Moreover, participants in the study may be younger (adult children) caregivers who are more familiar with the Internet as compared to older (spousal) caregivers. Another challenge is that the program is in English, and although many Indian people speak English, there is a low literacy rate (69.3% in 2011) [45] and it is certainly not the only language spoken in this country. This will result in the inclusion of a subgroup of caregivers that is highly educated [15, 46]. Another challenge may be that some households may not have reliable Internet coverage. Although this problem may decrease over time as the World Bank projects the greatest increases in Internet coverage to be in lower-middle income countries over the next few years, it may result in dropout during the study [47].

Anticipating challenges for inclusion and dropout, this study can be viewed as a first attempt to show the feasibility and potential of a self-help Internet-based training and support program for caregivers of people with dementia in a lower-resourced setting. A scalable approach is needed, given the large population of (adult child) caregivers of people with dementia, the urgent need for skills training and support to prevent and reduce caregiver distress, the growing number of Internet users, as well as the relatively low cost of such interventions [16, 18]. This also holds for other mental health interventions to reduce the mental health treatment gap [34, 48]. Internet-based interventions like iSupport may have potential for adaptation in differently resourced settings, and may help people with mental health problems around the world (Additional file 1).

## Trial status

The trial has completed initial planning and pilot testing. The trial is currently enrolling participants.

## Additional file

Additional file 1: SPIRIT 2013 Checklist: recommended items to address in a clinical trial protocol and related documents. (DOC 124 kb)

## Abbreviations

AD8: Alzheimer’s Disease Questionnaire-8-item
ARDSI: Alzheimer’s and Related Disorders Society of India
CES-D/CES-D10: Center for Epidemiologic Study Depression Scale
CTRI: Clinical Trials Registry—India
EOC: Education-only control
GAD-7: Generalized Anxiety Disorder scale-7-item
GEE: Generalized estimating equations
NIMHANS: National Institute of Mental Health and Neurosciences
RCT: Randomized controlled trial
T0: Pre measurement
T1: First follow-up
T2: Second follow-up
WHO: World Health Organization
ZBS: Zarit Burden Scale
